# Indigenous Bacteria Have High Potential for Promoting *Salix integra* Thunb. Remediation of Lead-Contaminated Soil by Adjusting Soil Properties

**DOI:** 10.3389/fmicb.2020.00924

**Published:** 2020-05-19

**Authors:** Xiaoyun Niu, Jian Zhou, Xiaona Wang, Xiaoyu Su, Shaohua Du, Yufei Zhu, Jinyu Yang, Dazhuang Huang

**Affiliations:** ^1^College of Landscape Architecture and Tourism, Hebei Agricultural University, Baoding, China; ^2^College of Forestry, Hebei Agricultural University, Baoding, China

**Keywords:** bacterial community, soil properties, Pb contamination, plant-microbe combined remediation, redundancy analysis

## Abstract

*Salix integra* Thunb., a fast-growing woody plant species, has been used for phytoremediation in recent years. However, little knowledge is available regarding indigenous soil microbial communities associated with the *S. integra* phytoextraction process. In this study, we used an Illumina MiSeq platform to explore the indigenous microbial composition after planting *S. integra* at different lead (Pb) contamination levels: no Pb, low Pb treatment (Pb 500 mg kg^–1^), and high Pb treatment (Pb 1500 mg kg^–1^). At the same time, the soil properties and their relationship with the bacterial communities were analyzed. The results showed that Pb concentration was highest in the root reaching at 3159.92 ± 138.98 mg kg^–1^ under the high Pb treatment. Planting *S. integra* decreased the total Pb concentration by 84.61 and 29.24 mg kg^–1^, and increased the acid-soluble Pb proportion by 1.0 and 0.75% in the rhizosphere and bulk soil under the low Pb treatment compared with unplanted soil, respectively. However, it occurred only in the rhizosphere soil under the high Pb treatment. The bacterial community structure and microbial metabolism were related to Pb contamination levels and planting of *S. integra*, while the bacterial diversity was only affected by Pb contamination levels. The dominant microbial species were similar, but their relative abundance shifted in different treatments. Most of the specific bacterial assemblages whose relative abundances were promoted by root activity and/or Pb contamination were suitable for use in plant-microbial combination remediation, especially many genera coming from *Proteobacteria*. Redundancy analysis (RDA) showed available nitrogen and pH having a significant effect on the bacteria relating to phytoremediation. The results indicated that indigenous bacteria have great potential in the application of combined *S. integra*-microbe remediation of lead-contaminated soil by adjusting soil properties.

## Introduction

Lead (Pb) is a widely distributed toxic element in soil with no biological function (Kushwaha et al., 2018). It is produced by mining and smelting activities, burning of leaded gasoline, as well as disposal of sewage sludge, batteries, and other lead-containing products (Huang et al., 2006). The accumulation of Pb causes serious harm to human, particularly on children. Many techniques to reduce its effects on ecosystems and humans have been tried, including physical, chemical, and biological processes, while most of them are high cost and also lead to secondary pollution (Ma et al., 2016). Combined microbial and plant remediation has become an popular way for its low cost, no secondary contamination, and being superior to single phytoremediation and microbiological methods (Khalid et al., 2017). Hence, plant-microbial remediation has become a research hotspot.

Although bacterially-assisted phytoremediation may seem pretty good at first for remediation of heavy metal contaminated soils, previous research has been performed only in pot culture and focused on the function of a single microbial inoculum (Hou et al., 2018; Zhang et al., 2018; Ramirez et al., 2019; Ju et al., 2020). They overlooked the complexity of the plant-soil environment in which abundant indigenous microbes may surpass or suppress such inoculated microbes (Wood et al., 2016; Kong et al., 2019; Xiao et al., 2020), and therefore the practical application of bacterially-assisted phytoremediation has generally been restricted (Xuliang et al., 2007; Kong et al., 2019; Xiao et al., 2020). However, mounting research has proved the importance of indigenous rhizosphere microbes in the process of phytoremediation (Thijs et al., 2016; Egamberdieva et al., 2017; Hou et al., 2019). A unique microbiome harboring in the rhizosphere of hyperaccumulator *Sedum alfredii* made a great contribution to the accumulation of trace metals (Hou et al., 2017a). Plants can accumulate more trace metals in natural soil than in γ-irradiated soil due to the differences in the rhizosphere microbial communities (Muehe et al., 2015; Hou et al., 2017b). Bacterial inoculation enhanced phytoremediation through interactions with indigenous rhizosphere bacteria (Hou et al., 2019; Joe et al., 2019). The study of the indigenous microbial community is, therefore, very important for phytoremediation, especially in the rhizosphere microenvironment.

Rhizosphere microbes are affected by soil physicochemical properties and heavy metal concentrations (Philippot et al., 2013; Guo et al., 2017; Lin et al., 2019; Cao et al., 2020). Soil organic matter, nitrogen, and pH can greatly influence the soil microbial structure and heavy metal content by affecting the bioavailability of heavy metals in soil (Lin et al., 2019; Wang et al., 2019a; Zhen et al., 2019; Cao et al., 2020). The soil N: P ratios and available phosphorus affected bacterial biodiversity and community composition in a reddish paddy soil (Huang et al., 2019). Soil Na^+^ significantly changes dominant microbes in Cd-contaminated soil (Wang et al., 2019b). Adjusting rhizosphere microbial community structure could, therefore be used to promote trace metal absorption by plants.

*Salix integra* Thunb., is a fast-growing woody species, with a large biomass and deep root system (Kuzovkina and Volk, 2009; Niu et al., 2019). It is a well-known Pb/Zn/Cd co-accumulator native to China (Liu et al., 2011). Previous studies have evaluated the ability of *S. integra* to absorb heavy metals such as Pb, Cu, Cd, and Zn (Yang et al., 2014; Shi et al., 2017; Cao et al., 2018, 2020; Yang et al., 2018). However, little is known regarding the indigenous rhizosphere microorganism during the phytoremediation by *S. integra*., which hinders the further application of microbial remediation with *S. integra*. This present study, therefore, investigates the characteristics of the indigenous bacterial community after phytoremediation by *S. integra* under different Pb levels using 16S rRNA gene amplicon sequencing as well as the interaction impacts of Pb contamination and root activity on the structure of soil bacterial communities. We aimed to (i) evaluate the effect of planting *S. integra* and Pb exposure on the indigenous bacterial community from different parts of the experiment (rhizosphere, bulk, and unplanted) in response to different Pb contamination levels, (ii) the effect of planting *S. integra* on Pb bioavailability from different parts of the experiment, and (iii) explore the key bacterial taxa involved in the phytoremediation process of Pb-contaminated soil, and their relationship with the soil properties.

## Materials and Methods

### Study Site and Plant Material

The experiment was conducted in Baoding experimental station of Hebei Agricultural University, Baoding City, Hebei Province, China, which has a temperate climate (38°45′21″ N, 115°24′37″ E) with mean annual temperature ∼13.0°C and annual precipitation about 532 mm. The soil is a typical meadow cinnamon soil in which the average concentration of Pb is 14.15 mg kg^–1^ lower than the soil background value (21.5 mg kg^–1^ in Hebei province). *S. integra* is a shrub of the family Salicaceae, and 1-year-old cuttings were selected for use with similar growth and vigor in this study.

### Experimental Set Up

**FIGURE 1:**
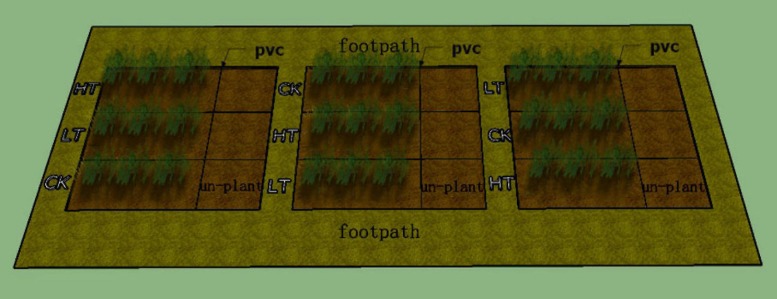
The layout of the study site.

The experiment was conducted in three square plots, 4 × 4 m and 0.6 m deep. This method was closer to the natural growth of the plant than pot experiments. Three Pb treatment regimens were conducted on each of the plots: 0 mg kg^–1^ (CK), low Pb treatment of 500 mg kg^–1^ (LT), and high Pb treatment of 1,500 mg kg^–1^ (HT). These correspond to the levels of Pb pollution found in urban soils and lead mines in China (Wu et al., 2003; Qin et al., 2019). Sheets of a perfluorinated ethylene-propylene copolymer plastic were put on the bottom and sides of the three plots, to prevent seepage. In each treatment, we planted *S. integra* and set out the unplanted area at the same time. They were separated by a PVC sheet in each plot. The layout of the study site is shown in Figure 1.

Pb was added into the soil in the form of Pb(NO_3_)_2_ solution in February 2017. The water volume was calculated by the maximum water-holding capacity, and the weight of Pb(NO_3_)_2_ was calculated by dry soil weight and treat concentration. After the powder of Pb(NO_3_)_2_ dissolving completely, it was sprayed and mixed with soil. The soil was plowed and mixed every 10 days, and were aged and equilibrated for 3 months under natural conditions (the soil moisture being at ∼75% of the maximum water-holding capacity), then the Pb concentration was measured. One-year-old cuttings were grown in May 2017. Eight cuttings were grown in each treatment. The awning with a transparent plastic sheet was constructed above the experiment area to avoid contaminating other soil when it rained. The control and treatments were all take the same agronomic management measures in the study period.

The initial soil properties were as follows: “organic carbon 2,780 mg kg^–1^, total nitrogen 240 mg kg^–1^, available nitrogen 22.28 mg kg^–1^, total phosphorus 510 mg kg^–1^, available phosphorus 10.54 mg kg^–1^, total potassium 8,080 mg kg^–1^, available potassium 278.4 mg kg^–1^, cation exchange capacity 8.46 cmol kg^–1^, and pH 8.14” (Niu et al., 2019). The Pb concentrations were as follows: total Pb concentrations were 14.21 mg kg^–1^, 510.28 mg kg^–1^, 1513.45 mg kg^–1^ and the acid-soluble Pb concentrations were 0.42 mg kg^–1^, 15.32 mg kg^–1^, 81.52 mg kg^–1^ in CK, LT and HT, respectively.

### Soil Sampling and Plant Harvesting

After 17 months growth (in September 2018), samples of the plants, rhizospheric soil, bulk soil, and unplanted soil were collected in three replicates under each treatment. It is important to note that the aerial parts of the plant were ever reaped in November 2017. The plants were completely uprooted and shaken gently. After that, the soil adhering to the root was collected by brush as the rhizosphere soil sample. The soil from five plant roots was pooled as a mixed sample of rhizospheric soil. The bulk soil and unplanted soil were all obtained by soil-drilling at a depth of 20–30 cm, and five random spots were mixed as one replicate. Approximately 3 g soil from each replication was stored at −80°C until DNA extraction. Residual soils were air dried to measure soil physicochemical properties and Pb content. When sampling, all tools were sterilized in different treatments.

At harvest, roots, trunk, branch, and leaves were harvested separately, then rinsed thoroughly with water, and finally rinsed with deionized water. The samples were through drying at 65°C, weighing, grinding, and passing through a 60 mesh sieve to measure Pb content.

### Soil Analyses

After the soil drying and passing through a 10 mesh sieve, the soil properties were measured, such as available nitrogen (AN), available phosphorus (AP), available potassium (AK), pH, and cation exchange capacity (CEC), following the methods of Niu et al. (2019).

### Total Pb Content in Plant Tissues and Soils

0.5 g of the powdered root, branch, leaf or soil was digested in a mixture of HNO_3_ and HClO_4_ (10:1, v/v, 10 mL) by microwave digestion (Sined, MDS-6, Shanghai, China). The mixture was digested at 180°C for about 3.5 h, until the solution become clarification. It was diluted to 50 mL, and filtered by 0.45 μm membrane. Total Pb in the extractant was measured by atomic absorption spectrophotometry (AA-680, SHIMADZU, Kyoto, Japan).

### Fractionation of Pb in Soil

Partitioning of Pb was measured by the sequential extraction procedure of Rauret et al. (1999), including the “acid-soluble,” “reducible,” “oxidizable,” and “residual” fractions, using 0.5 g soil. The extractions were as follows: acid-soluble extraction using acetic acid (0.11 mol L^–1^, 16 h), reducible extraction using hydroxylamine hydrochloride (0.5 mol L^–1^, pH = 1.5, 16 h), oxidizable extraction using H_2_O_2_ (8.8 mol L^–1^, 2 × 1 h, 85°C) and then following extraction using ammonium acetate 1.0 mol L^–1^. The digestion of residual fraction was through adding three acids (4 mL HCl, 2 mL HNO_3_, and 2 mL HF). The different forms of Pb were measured by atomic absorption spectrophotometry.

### DNA Extraction, Bacterial 16S Amplification, and MiSeq Sequencing

It is important to note that we measured only rhizosphere and bulk soil samples in CK and the low Pb treatment (LT), but measured rhizosphere, bulk, and unplanted soils in the high Pb treatment (HT). The PowerSoil DNA isolation kit (MOBIO, San Diego, United States) was used to extract soil genomic DNA from 0.5 g soil following the manufacturer’s instructions, and a NanoDrop 2000 spectrophotometer (Thermo Fisher Scientific, Waltham, United States) was used to measure DNA concentration. The V3-V4 regions of the bacterial 16S rRNA genes were amplified using the primer set DBV34F (5′-GTACTCCTACGGGAGGCAGCA-3′) and DBV34R (5′-GTGGACTACHVGG GTWTCTAAT-3′). All PCR amplifications were conducted in triplicate with a total reaction volume of 25 μL containing 1 μL (5 μmol L^−1^) of each forward/reverse primer, 30 ng genomic DNA, BSA 3 μL (2 ng μL^–1^), 2 × Taq Master Mix 13.5 μL (Allwegene Biotech Co., Ltd., Beijing, China). The PCR was carried out under the conditions: initial denaturation: 94°C, 5 min; 25 cycles (denaturation: 94°C, 30 s; annealing: 50°C, 30 s; and extension: 72°C, 60 s); with a final extension: 72°C, 7 min. PCR amplicons were purified using a Purification Kit (Qiagen, Shenzhen, China), and the concentration of purification was measured by a NanoDrop 2000 spectrophotometer. Equivalent PCR products from all repeats were combined into a mixed sample, and sequenced using a MiSeqPE300 platform (Illumina, United States; Allwegene Biotech Co., Ltd., Beijing, China).

### Bioinformatic Analysis of 16S rRNA Gene Sequences

QIIME v1.7.0 was used to de-multiplex, quality filter, and analyze raw Illumina fastq files (Caporaso et al., 2012). Reads were filtered with less than 200 bp long or 25 of the average quality score. Chimeric sequences were removed using Chimera and filtered by USEARCH (Edgar et al., 2011), and singletons were also removed. Operational taxonomic units (OTU) were divided by UCLUST according to 97% similarity threshold (Edgar, 2013). Phylogenetic classification of the reads was identified according to the Ribosomal Database Project (RDP), following 80% threshold (Wang et al., 2007). The classification of representative OTUs were according to similarity to the SILVA (release_132) database. OTUs belonging to plants were removed. In order to decrease the effects induced by sequencing depth, all samples were rarefied to the same sequence depth. Shannon, Chao 1, and Observed species were used to estimate the alpha microbial biodiversity (Pagaling et al., 2014).

### Statistical Analyses

Each data point represented the results of three replications performed as average value ± standard error. The effects of Pb contamination levels and planting *S. integra* on different treatments were tested by a two-way ANOVA. The Shapiro-Wilk test was used to check the analyzed features’ normality. The significant differences were checked by the least significant difference (LSD) in the parameter test or the Kruskal-Wallis test in the non-parameter according to the distribution of the estimated parameters. In all analyses, *p-*value < 0.05 meant significant difference. The package SPSS 19.0 (IBM, America) was used to analyze the data. Redundancy analysis (RDA) was conducted to test the relationship of soil properties and bacterial communities performed by the software CANOCO (Windows version 4.5) (Biometris-Plant research international, Wageningen, the Netherlands). Non-metric multidimensional scaling (NMDS) was used to visualize differences based on community composition by the metaMDS function of the vegan package in *R* package. The microbial metabolism heat map and cluster analyses were conducted, based on relative abundance and Bray-Curtis distances, with the agnes function of the cluster package in *R* package.

## Results

### Effect of Pb on Plant Growth and Heavy Metal Uptake

Plants were exposed to the Pb-polluted and unpolluted soil for 17 months and appeared significant difference in biomass (Supplementary Table S1). On the whole, the growth of *S. integra* was not inhibited in the low Pb treatment (Pb500). The plant biomass in CK (Pb 0) and the low Pb treatment had no significant difference, but both were higher than in the high Pb treatment (Pb1500). The total amount of Pb uptake by *S. integra* was highest in the high Pb treatment, followed by the low Pb treatment. Pb concentration was highest in the root, reaching 1221.36 ± 111.36 mg kg^–1^ in the low Pb treatment and 3159.92 ± 138.98 mg kg^–1^ in the high Pb treatment, followed by the trunk and leaf, and lowest in the branch whether in CK, low Pb treatment, or high Pb treatment. It may be contributed to the lower transfer factor from the root part to the aerial part. The Pb concentrations in *S. integra* planted in Pb-treated soils were significantly greater than that planted in the control soils. Heavy metal accumulation in the plant was related to heavy metal concentration in soil. The Pb accumulation of *S. integra* grown in soil containing 1,500 mg kg^–1^ Pb was 1.98 times as much as that grown in soil containing 500 mg kg^–1^ Pb. The results got here clearly show that *S. integra* has great potential to tolerate high Pb concentrations.

### Soil Chemical Properties

The contents of available potassium and available nitrogen were affected by the interaction of Pb contamination level with soils from different parts of the experiment (rhizosphere, bulk, and unplanted), while available phosphorus and CEC were only affected by Pb contamination or by differences in the soil samples (Supplementary Figures S1A–E). Overall, Pb contamination had a negative effect on CEC, available potassium, and available phosphorus, and had a positive effect on available nitrogen. However, root activity promoted them all, except CEC.

### Pb Content in Rhizosphere, Bulk, and Unplanted Soil After Phytoremediation

**FIGURE 2:**
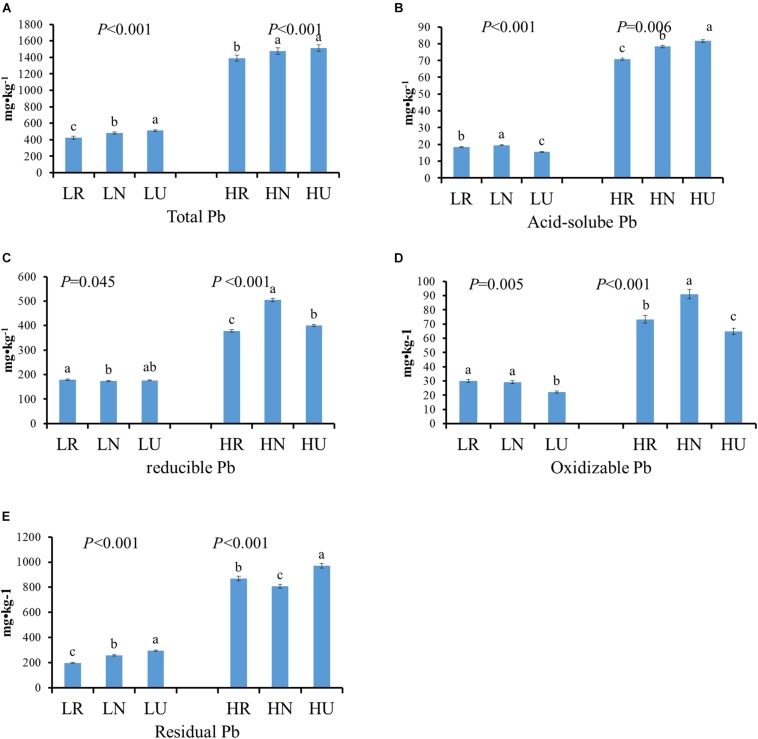
Different fractions Pb content in rhizosphere, bulk, and unplanted soil after phytoremediation. **(A)** Total Pb. **(B)** Acid-soluble Pb. **(C)** Reducible Pb. **(D)** Oxidizable Pb. **(E)** Residual Pb. LR, rhizosphere soil in the low Pb treatment; LN, bulk soil in the low Pb treatment; LU, unplanted soil in the low Pb treatment; HR, rhizosphere soil in the high Pb treatment; HN, bulk soil in the high Pb treatment; HU, unplanted soil in the high Pb treatment. Different letters indicate that the values differ significantly at p < 0.05.

The total Pb concentration after the phytoremediation decreased by 84.61 mg kg^–1^ in rhizosphere and 29.24 mg kg^–1^ in bulk soil in the low Pb treatment (LT), but it occurred only in the rhizosphere soil in the high Pb treatment (HT) (Figure 2A). However, there was no significant difference between bulk and unplanted soil in the high Pb treatment. Four different fractions of Pb from soil samples were extracted and analyzed (Figures 2B–E). Phytoremediation decreased the acid-soluble Pb concentration of planted soil in the high Pb treatment, but showed the opposite effect on the low Pb treatment. It increased the concentration of oxidizable Pb and decreased the concentration of residual Pb in planted soil compared with unplanted soil. The concentration of acid-soluble Pb, oxidizable Pb, and residual Pb were all higher in bulk than in rhizosphere soil, except residual Pb in the high Pb treatment. The concentration of reducible Pb showed no significant difference between planted soil and unplanted soil in the low Pb treatment. However, it was higher in the bulk and lower in rhizosphere soil compared with unplanted soil in the high Pb treatment, respectively.

### Partitioning of Pb in Rhizosphere, Bulk, and Unplanted Soil After Phytoremediation

**FIGURE 3:**
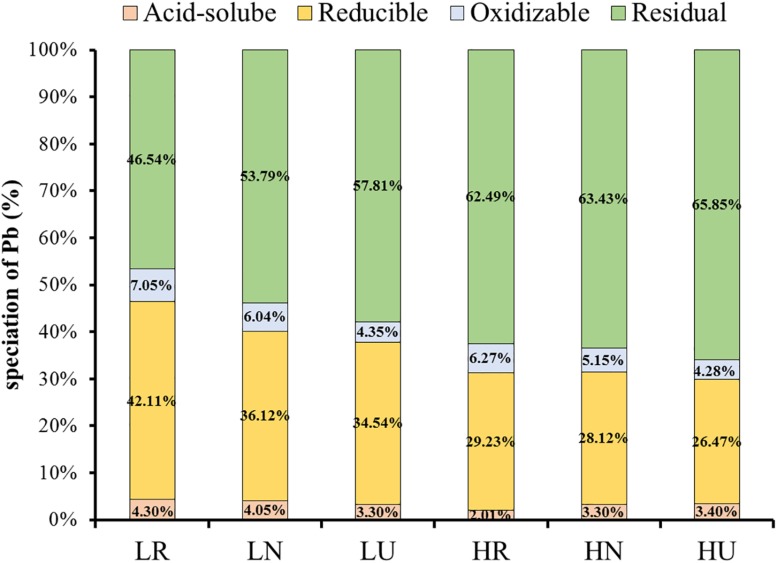
Effect of *S. integra* phytoremediation on speciation of Pb (%) in the low and high Pb treatments. LR, rhizosphere soil in the low Pb treatment; LN, bulk soil in the low Pb treatment; LU, unplanted soil in the low Pb treatment; HR, rhizosphere soil in the high Pb treatment; HN, bulk soil in the high Pb treatment; HU, unplanted soil in the high Pb treatment. Different letters indicate that the values differ significantly at *p* < 0.05.

The ratio of Pb under four fractionations was similar in different samples (Figure 3). Quite a large amount of residual and reducible Pb was found in the soil, but very little oxidizable and acid-soluble Pb. Planting *S. integra* promoted the proportion of bioavailable fractions in the low Pb treatment, but not in the high Pb treatment. Compared with the unplanted soil, the acid-soluble Pb proportion in the rhizosphere and bulk soil was increased by 1.0 and 0.75%, respectively, and the residual Pb proportion was decreased in the low Pb treatment. Furthermore, the acid-soluble Pb proportion of the rhizosphere soil was highest in the low Pb treatment, but decreased in the high Pb treatment when compared with the unplanted soil. The acid-soluble Pb proportion was higher in the low Pb treatment than in the high Pb treatment, whether in the rhizosphere or bulk soil, while the residual Pb proportion showed the opposite trend. The reducible and oxidizable Pb proportion showed a similar trend, and they were all promoted by phytoremediation in the rhizosphere and bulk soil when compared with unplanted soil.

### Sequencing Summary of the Pyrosequencing Data and Alpha Diversity Indices

The high-quality reads generated by 16S rRNA gene amplicon sequencing were classified according to a 97% sequence identity threshold, which produced 5,505 bacterial operational taxonomic units (OTUs). A total of 5,505 different OTUs belonged to 660 genera, 396 families, 257 orders, 117 classes, and 41 phyla. Of these OTUs, only 0.1% belonged to archaea. The coverage rang were between 94.45 and 95.57%, showing that most of the bacterial taxa were detected in the soil samples (Table 1).

**TABLE 1:** Summary of the pyrosequencing data and alpha diversity indices.

Index	Treatments						
	CR	CN	LR	LN	HR	HN	HU
Total raw tags	194,996	152,915	256,015	192,393	198,422	172,903	143,422
No. of OUT	2865.33 ± 123.45	2806.33 ± 62.13	2739.33 ± 16.25	2754.66 ± 32.02	2561.33 ± 35.50	2580 ± 44.17	2657 ± 94.72
No. of phylum	28 ± 0.73	30 ± 1.2	29 ± 1.62	29 ± 0.25	26 ± 1.78	30 ± 2.13	29 ± 1.25
No. of class	86 ± 2.56	81 ± 3.21	90 ± 3.0	86 ± 1.56	82 ± 1.45	84 ± 2.12	88 ± 2.41
No. of order	227 ± 5.26	230 ± 6.58	231 ± 4.58	232 ± 4.89	230 ± 3.56	221 ± 3.12	236 ± 4.25
No. of family	244 ± 3.26	246 ± 5.26	250 ± 5.42	237 ± 3.65	239 ± 3.24	235 ± 4.58	256 ± 5.12
No. of genus	360 ± 4.26	340 ± 5.23	359 ± 6.23	353 ± 4.26	345 ± 5.89	358 ± 6.48	325 ± 5.74
Chao1	3823.29 ± 113.26	3822.51 ± 92.21	3728.72 ± 76.33	3754.08 ± 34.72	3538.95 ± 12.98	3551.97 ± 123.80	3620.19 ± 82.11
Observed species	2759.63 ± 121.03	2699.3 ± 60.06	2637.76 ± 19.96	2647.36 ± 35.95	2462.26 ± 38.03	2480.26 ± 38.25	2553.4 ± 91.01
Shannon	9.85 ± 0.12	9.72 ± 0.07	9.66 ± 0.004	9.73 ± 0.03	9.44 ± 0.08	9.46 ± 0.06	9.53 ± 0.11
Coverage (%)	95.12 ± 2.12	94.45 ± 2.47	95.57 ± 3.12	94.25 ± 3.54	95.54 ± 2.58	94.32 ± 3.78	94.47 ± 2.89

*CR, rhizosphere soil in CK; CN, bulk soil in CK; LR, rhizosphere soil in the low Pb treatment; LN, bulk soil in the low Pb treatment; HR, rhizosphere soil in the high Pb treatment; HN, bulk soil in the high Pb treatment; HU, unplanted soil in the high Pb treatment.*

### Alpha-Diversity of the Bacterial Community and Venn Diagram

The observed species, Chao 1, and Shannon index were used to compare alpha-diversity of the bacterial community among different samples, and they were only significantly influenced by Pb contamination levels (Supplementary Figure S2A). Chao 1 appeared decreasing trend with increase of Pb levels regardless of sampling from different parts of the experiment. However, observed species and Shannon’s index were only decreased in the high Pb treatment, and showed no significant difference between the CK and low Pb treatments. The shared genera of bacterial communities were performed by Venn diagrams among samples (Supplementary Figure S2B). The vast majority of the genera were common to all samples, which was likely caused by the same soil source and similar experimental conditions.

### Bacterial Community Structure

**FIGURE 4:**
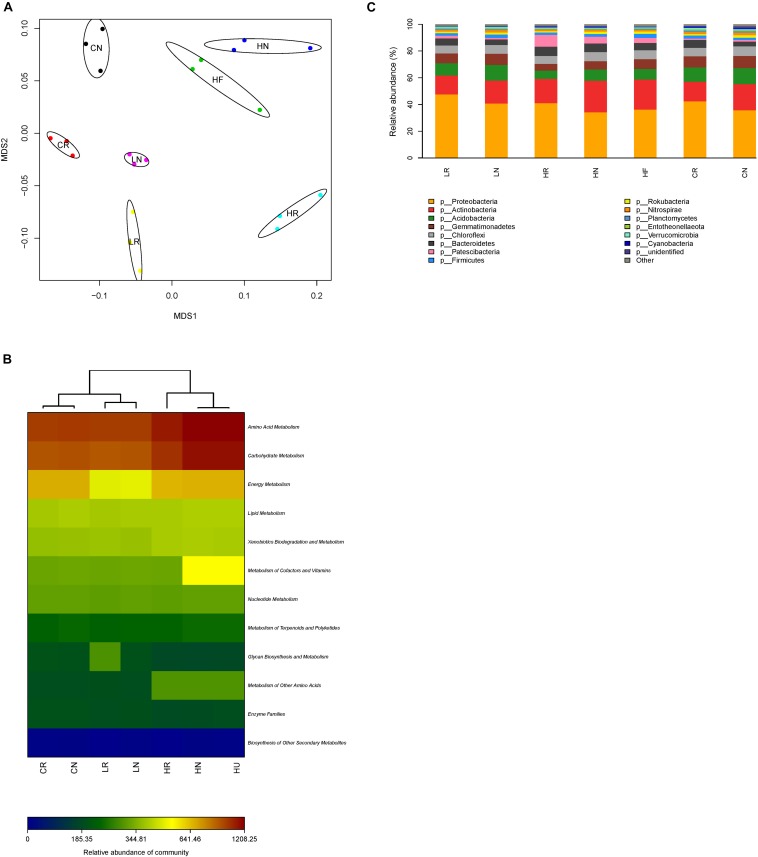
Bacterial community structure. **(A)** Non-metric multidimensional scaling (NMDS) ordination plots derived from Unifrac distance matrix, **(B)** microbial metabolism heat map, and **(C)** the relative abundance of bacteria in rhizosphere, bulk, and unplanted soil with different treatments at phylum level. CR, rhizosphere soil in CK; CN, bulk soil in CK; LR, rhizosphere soil in the low Pb treatment; LN, bulk soil in the low Pb treatment; HR, rhizosphere soil in the high Pb treatment; HN, bulk soil in the high Pb treatment; HU, unplanted soil in the high Pb treatment.

The non-metric multidimensional scaling (NMDS) analysis revealed a clear differentiation of bacterial community structure (Figure 4A). The bacterial community composition of rhizosphere, bulk, and unplanted soil samples from different Pb contaminations formed distinct clusters in the NMDS ordination plots, implying that the bacterial community structure was affected by the root activity and Pb contamination. To provide a functional understanding of the microbial community in the different samples, the metabolic functions were predicted by the software Picrust and the KEGG database (Langille et al., 2013). From the metabolism heat map (Figure 4B), we can see that microbial metabolism has significant differences in different samples, especially amino acid metabolism and carbohydrate metabolism. They were also clustered in relation to the Pb contamination levels and root activity. The microbial metabolism of the low Pb treatment was more similar to CK.

The sequencing reads obtained from all samples classified at the phylum level were attached to 14 bacterial phyla (Figure 4C). The dominant phyla with relative abundance more than 1% of the overall community in each sample were similar, while the relative abundance shifted. The relative abundance of *Proteobacteria* was highest, and was increased by root activity which was higher in rhizosphere soil, and promoted in the low Pb treatment compared with CK (Table 2). However, *Actinobacteria* showed the opposite trend, being higher in bulk soil, and improved in the high Pb treatment compared with CK. The interaction of soils from different parts of the experiment and Pb contamination had a significant effect on *Acidobacteria* and *Patescibacteria* (Table 2). Overall, *Acidobacteria* was decreased in the high Pb treatment regardless of whether in rhizosphere soil, bulk soil, or unplanted soil, but not in the low Pb treatment. *Patescibacteria* was increased only in the high Pb treatment and higher in the bulk soil. Both the relative abundance of *Gemmatimonadetes* and *Chloroflexi* were higher in the bulk soil, while only *Gemmatimonadetes* was decreased by Pb contamination. *Bacteroidetes* was higher in the bulk soil and increased in the high Pb content (Table 2).

**TABLE 2:** The relative abundance of main phyla in different samples.

Phyla	*Proteobacteria*	*Actinobacteria*	*Acidobacteria*	*Gemmatimonadetes*	*Chloroflexi*	*Bacteroidetes*	*Patescibacteria*
CR			0.09 ± 0.02ab				0.012 ± 0.0005c
CN			0.12 ± 0.02a				0.012 ± 0.0021c
LR			0.12 ± 0.02a				0.019 ± 0.0078c
LN			0.11 ± 0.001ab				0.011 ± 0.00007c
HR			0.06 ± 0.01c				0.107 ± 0.0047a
HN			0.08 ± 0.02bc				0.033 ± 0.0013b
HU			0.08 ± 0.01bc				
R	0.43 ± 0.038a	0.15 ± 0.02b		0.068 ± 0.015b	0.061 ± 0.005b	0.060 ± 0.01a	
N	0.37 ± 0.049b	0.19 ± 0.04a		0.078 ± 0.012a	0.068 ± 0.006a	0.046 ± 0.017b	
CK	0.39 ± 0.042b	0.17 ± 0.03b		0.087 ± 0.004a		0.046 ± 0.015b	
LT	0.45 ± 0.037a	0.15 ± 0.02b		0.077 ± 0.006b		0.046 ± 0.008b	
HT	0.37 ± 0.041b	0.21 ± 0.04a		0.056 ± 0.007c		0.066 ± 0.013a	
*P* (PbC)	0.001	0.001	0.099	<0.001	0.015	0.021	
*P* (DCS)	0.001	0.001	0.044	<0.001	0.317	0.009	
*P* (PbC*DCS)	0.591	0.427	0.004	0.401	0.924	0.309	

CK, Pb 0 mg kg^−1^; LT, Pb 500 mg kg^−1^; HT, Pb 1500 mg kg^−1^; R, rhizosphere soil; N, bulk soil; CR, rhizosphere soil in CK; CN, bulk soil in CK; LR, rhizosphere soil in the low Pb treatment; LN, bulk soil in the low Pb treatment; HR, rhizosphere soil in the high Pb treatment; HN, bulk soil in the high Pb treatment; HU, unplanted soil in the high Pb treatment. PbC, Pb contamination effect; DCS, soils from different parts of the experiment; PbC*DCS, the interactive effect of Pb contamination and soils from different parts of the experiment. Different letters indicate that the values differ significantly at *p* < 0.05. Values represent mean ± SD (*n* = 3).

### Specific Bacterial Assemblages in Different Samples

The metastasis were used to analyze the specific bacterial assemblages whose relative abundance showed significant changes between rhizosphere and bulk soil in different treatments (Table 3). There were 88, 62, and 91 specific bacterial assemblages at a general level in CK, the low Pb treatment, and the high Pb treatment, respectively. In order to measure the effect of Pb contamination and root activity on the relative abundance of specific bacterial assemblages, two-way ANOVA was used to test 17 general bacteria that emerged in the top 10 microbes of relative abundance in each sample. From Tables 3A,B, we can see that *Lysobacter*, *Pseudomonas*, *Allorhizobium*, *Steroidobacter*, *Skermanella*, and *Acidovorax* were affected by the interaction of Pb contamination and root activity. The others were affected by Pb contamination and/or root activity. Overall, the relative abundances of partial bacterial genera were increased by Pb contamination and root activity; examples were *Lysobacter*, *Pseudomonas*, *Allorhizobium*, *Acidovorax*, and *Ensifer*, especially *Pseudomonas* and *Ensifer* which increased to more than 1%, while others were decreased by Pb contamination, such as *Steroidobacter* and *Skermanella*. Pb contamination had no significant effect on the relative abundance of *Nitrospira*, *Ramlibacter*, *Microvirga*, *Ellin6067*, and MND1 in the low Pb treatment, but had a positive effect in the high Pb treatment. In addition, root activity also had a positive effect on the relative abundance of *Ramlibacter*, *Microvirga*, and MM2. The relative abundances of *uncultured_bacterium* and *uncultured* were higher in all the treatments, while they were uncultured and not promoted by the root activity.

**TABLE 3A:** The relative abundance of specific bacterial assemblages.

		*Uncultured bacterium*	*Uncultured*	*Ensifer*	*Nitrospira*	*Ramlibacter*	*Microvirga*	*Ellin6067*	*MND1*	*Gaiella*	*MM2*
Pb	CK	15.99 ± 2.66b	14.32 ± 0.66a	0.59 ± 0.19b	1.62 ± 0.23a	0.48 ± 0.21a	0.5 ± 0.08a	0.5 ± 0.07a	4.33 ± 0.80a		
	LT	15.17 ± 1.20ab	12.98 ± 0.71b	0.93 ± 0.37b	1.54 ± 0.26a	0.49 ± 0.14a	0.43 ± 0.08a	0.44 ± 0.08a	3.92 ± 0.60a		
	HT	18.09 ± 1.86a	12.63 ± 0.67b	1.34 ± 0.31a	1.05 ± 1.13b	0.27 ± 0.13b	0.29 ± 0.07b	0.29 ± 0.07b	2.46 ± 0.42b		
Compartment	R	14.83 ± 1.61b	12.98 ± 1.15b	1.19 ± 0.39a	1.26 ± 0.22b	0.55 ± 0.16a	0.54 ± 0.11a			0.84 ± 0.15b	0.55 ± 0.30a
	N	18.00 ± 1.65a	13.64 ± 0.70a	0.72 ± 0.32b	1.56 ± 0.36a	0.28 ± 0.10b	0.44 ± 0.09b			1.25 ± 0.14a	0.10 ± 0.06b
PbC	*P*	<0.001	0.035	<0.001	<0.001	<0.001	0.003	0.747	0.057	<0.001	<0.001
DCS	*P*	<0.001	0.001	<0.001	<0.001	0.001	<0.001	0.001	<0.001	0.538	0.301
PbC*DCS	*P*	0.093	0.266	0.1	0.051	0.468	0.189	0.079	0.088	0.415	0.15

**B**											

		***Lysobacter***	***Pseudomonas***	***Allorhizobium***	***Steroidobacter***	***Skermanella***	***Acidovorax***	***Hydrogenophaga***			

Treatment	CR	0.35 ± 0.08c	0.2 ± 0.03b	0.28 ± 0.12b	1.48 ± 0.23a	1.17 ± 0.27a	0.11 ± 0.03c	0.20 ± 0.03b			
	CN	0.34 ± 0.01c	0.11 ± 0.02b	0.14 ± 0.03c	0.86 ± 0.13c	0.73 ± 0.09c	0.02 ± 0.01cd	0.11 ± 0.02b			
	LR	0.53 ± 0.04b	1.25 ± 0.59a	0.66 ± 0.07a	1.15 ± 0.10b	0.94 ± 0.13ab	0.82 ± 0.09a	1.25 ± 0.59a			
	LN	0.39 ± 0.004c	0.25 ± 0.03b	0.19 ± 0.03bc	1.17 ± 0.12b	1.08 ± 0.12a	0.11 ± 0.04c	0.25 ± 0.03b			
	HR	0.52 ± 0.07b	1.10 ± 0.37a	0.61 ± 0.04a	0.82 ± 0.10c	0.68 ± 0.06c	0.24 ± 0.06b	1.11 ± 0.37a			
	HN	1.09 ± 0.09a	0.09 ± 0.07b	0.14 ± 0.06c	0.72 ± 0.06c	0.69 ± 0.05c	0.006 ± 0.01d	0.09 ± 0.07b			
PbC	*P*	<0.001	0.002	<0.001	0.003	0.184	<0.001	<0.001			
DCS	*P*	<0.001	0.052	<0.001	<0.001	0.004	<0.001	0.009			
PbC*DCS	*P*	<0.001	0.041	0.001	0.003	0.009	<0.001	0.025			

CK, 0 mg kg^−1^; LT, Pb 500 mg kg^−1^; HT, Pb 1500 mg kg^−1^; CR, rhizosphere soil in CK; CN, bulk soil in CK; LR, rhizosphere soil in the low Pb treatment; LN, bulk soil in the low Pb treatment; HR, rhizosphere soil in the high Pb treatment; HN, bulk soil in the high Pb treatment. PbC, Pb contamination effect; DCS, soils from different parts of the experiment; PbC*DCS, the interactive effect of Pb contamination and soils from different parts of the experiment. Different letters indicate that the values differ significantly at *p* < 0.05. Values represent mean ± SD (*n* = 3).

### Relationships of Specific Bacterial Assemblages and Soil Properties

**FIGURE 5:**
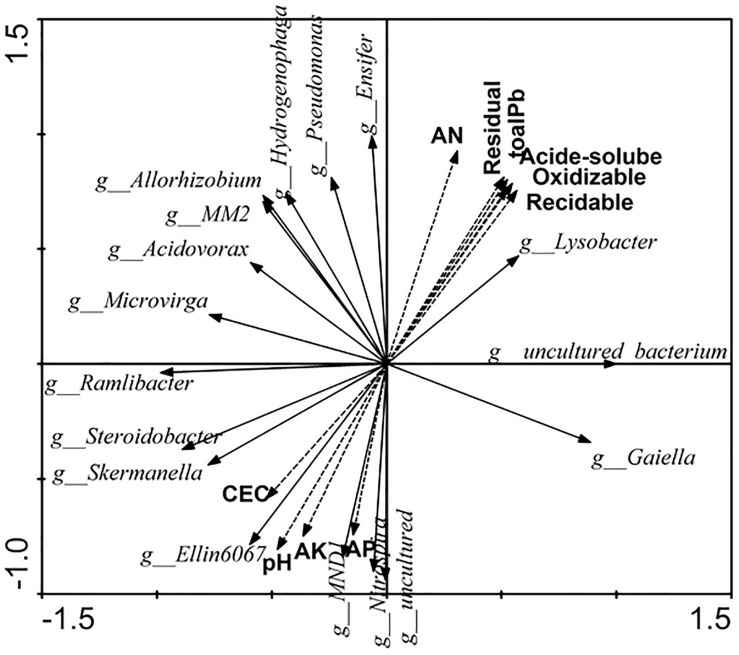
The relationships of soil properties and significance change bacterial of the relative abundance by Redundancy analysis. AN, available nitrogen; AK, available potassium; AP, available phosphorus.

The relationships of soil properties and specific bacterial assemblages were measured by redundancy analysis (RDA) (Figure 5). Eigenvalues of RDA showed that axes 1 and 2 explained 65.7 and 22.1% of the variance of the microbial quantities data, respectively. Available nitrogen (AN) and pH were significant (*F* = 5.315, *p* = 0.008 and *F* = 3.755, *p* = 0.048, respectively) in explaining the relative abundance of specific bacterial assemblages, and could account for up to 14.9 and 12.6%, respectively. The contributions of other factors to the observed variation were as follows: residual Pb 12.5%, acid-soluble Pb 12.4%, total soluble Pb 12.2%, reducible Pb 11.7%, oxidizable Pb 11.8%, available potassium 10.1%, CEC 6.5%, and available phosphorus 9.8%.

From Figure 5, we can see that *Lysobacter*, *Pseudomonas*, *Allorhizobium*, *Acidovorax*, and *Ensifer*, which were promoted by Pb and root activity, had a strong positive correlation with the available nitrogen and Pb content, and had a negative correlation with CEC, pH, available potassium, and available phosphorus. Furthermore, *uncultured bacterium*, which was the dominant genus, also had a strong correlation with the available nitrogen and Pb content. *Lysobacter*, *Pseudomonas*, *Allorhizobium*, *Acidovorax*, *Ensifer*, *Ramlibacter*, *Microvirga*, and *MM2* had stronger correlations with each other.

## Discussion

*Salix integra* has been reported to be potentially valuable for phytoremediation of Pb contaminated soils (Shi et al., 2017; Cao et al., 2018, 2019), which is supported by the results of our study (Supplementary Table S1 and Figure 2). However, the potential of *S. integra* for heavy metal extraction relies not only on its fast-growth, large biomass and deeper, more integrated root systems and powerful tolerance to Pb, but also on the underground processes that promote root uptake of the heavy metal. A high-throughput sequencing approach was therefore used to measure rhizospheric bacterial community that may influence the accumulation of heavy metal in *S. integra.*

Planting *S. integra* decreased the total Pb concentration both in rhizosphere and bulk soil in the low Pb treatment, which could be explained by the effects of root activity not being limited to the root surface, though the effect declined when the distances increase (Yang et al., 2017). Furthermore, our previous study also showed that microbial quantity and metabolism were promoted both in the rhizosphere and bulk soil in the low Pb treatment (Niu et al., 2019). However, it only occurred in the rhizosphere soil under high Pb treatment. The efficiency of phytoextraction relies greatly on the bioavailability of heavy metals. The concentration and proportion of acid-soluble Pb with the highest bioavailability were not decreased for *S. integra* uptake but increased in planted soil when compared with unplanted soil in the low Pb treatment, which may be attributed to higher transformation rate than uptake for the acid-soluble fraction. However, the opposite trend appeared in high Pb treatment. The important components of root exudates, such as organic and amino acids can promote the transformation of acid-soluble Pb from other species of the heavy metals in the low Pb treatment (Luo et al., 2017, 2019, 2020; Liu et al., 2018; Moshiri et al., 2019). However, the plant root may exude carboxyl groups and phenols to inhibit the transformation of acid-soluble Pb due to its high toxicity, and thus protect themselves in the high Pb treatment (Liu et al., 2017; Moshiri et al., 2019).

The soil microbes alter their structure to acclimatize different levels of heavy metal pollution and root environments, which is consistent with the previous studies (Edwards et al., 2015; Li et al., 2017; Liu et al., 2018; Lin et al., 2019; Cao et al., 2020). The shift of microbial composition led to metabolic function variation (Tipayno et al., 2018; Liu et al., 2020), and further caused Pb speciation differences in different treatments. It could explain the difference of acid-soluble Pb concentration between the low and high Pb treatments. Heavy metal pollution inhibits the metal-sensitive species, and stimulates metal-resistant species, microbial diversity therefore affects (Xie et al., 2016; Cao et al., 2020). Bacterial diversity was not affected by root activity, contrary to the rhizosphere bacterial community associated with *Sedum alfredii* (Hou et al., 2018), and that may result from the differences of plant root and soil environment.

Dominant bacterial species were similar among different treatments, which may be related to the same soil environment (Fan et al., 2016), and their relative abundances shifted that contributed to the selection of root activity and Pb contamination. Most of the specific bacterial assemblages between rhizosphere and bulk soil were promoted by root activity and/or Pb contamination, and are suitable for use in plant-microbial combination remediation, for example *Proteobacteria, Actinobacteria, Patescibacteria, Bacteroidetes*, and *Gemmatimonadetes.* The relative abundance of *Proteobacteria* was the highest of any treatment (more than 35%), which were also reported to be the dominant phyla in soils contaminated by polycyclic aromatic hydrocarbons, Cr, Cu, Pb, and Zn, always exceeding 50% in Pb contaminated soils (Bourceret et al., 2016; Fan et al., 2016; Tipayno et al., 2018; Lin et al., 2019). We, therefore, infer that it plays an important role in phytoremediation by *S. integra. Actinobacteria* are also increased after phytoremediation by *Sedum* in Cd-contaminated soil (Hou et al., 2018). Many of them can produce IAA to stimulate plant growth, as well as siderophores to form stable complexes with heavy metals (Rajkumar et al., 2010). *Patescibacteria* and *Bacteroidetes* have been reported as dominant phyla in heavy metal-polluted soils (Ellis et al., 2003; Idris et al., 2004), although their relationship with root activity was small in our study. *Gemmatimonadetes* was decreased by Pb contamination, but Hou et al. (2018) found it was positively associated with Cd levels. These conflicting results may be caused by the difference in the metal levels and soil physicochemical properties and the history of contamination in the different studies (Fan et al., 2016).

At a general level, partial bacterial genera, such as *Lysobacter*, *Pseudomonas*, *Allorhizobium*, *Acidovorax*, and *Ensifer* were increased by Pb contamination and root activity. Among them, *Pseudomonas* and *Ensifer* which increased to more than 1% played key roles during phytoremediation by *S. integra.* Moreover, *Pseudomonas* strains have been developed as bioinoculants for phytoremediation, bioremediation of metals, and methylmercury degradation (Boeris et al., 2016; Cabral et al., 2016; Sun et al., 2017). *Uncultured bacteria* with higher abundance in all the treatments were promoted only by Pb contamination, but their functions cannot be ignored. From all of these observations, we can infer that indigenous soil microbial communities played very important roles in promoting heavy metals absorption by *S. integra.*

In this study, the specific bacterial assemblages were promoted by available nitrogen and decreased by high pH, which is consistent with previous studies (Guo et al., 2019; Huang et al., 2019; Wang et al., 2019a; Zhen et al., 2019). Available nitrogen and available phosphorus are a significant energy source for microbes and play an important role in regulating their adaptation to different levels of heavy metal contamination (Guo et al., 2017; Lin et al., 2019). We also found that the specific bacterial assemblages, such as *Pseudomonas*, *Allorhizobium*, *Acidovorax*, *Ensifer*, *Ramlibacter*, *Microvirga*, and *MM2*, all had stronger correlations, which implied that plant-microbe combined remediation cannot rely on only a kind of microbe. We could, therefore, shift microbial composition by adjusting soil properties, for example adding available nitrogen, so promoting *S. integra* absorption of Pb.

## Conclusion

During phytoremediation, the relative abundance of dominant microbial species shifted. The bacteria were promoted by root activity and/or Pb contamination, especially many genera from *Proteobacteria*, which are suitable for using in plant-microbial combination remediation. Indigenous bacteria have great potential, therefore, in the application of combined *S. integra*-microorganism remediation of lead-contaminated soil by adjusting soil properties, for example available nitrogen and pH. Further study on promoting plant-indigenous bacteria combined remediation through by adjusting soil properties and its practical application will be conducted.

## Data Availability Statement

The datasets generated for this study can be found in https://www.ncbi.nlm.nih.gov/sra/PRJNA588741.

## Author Contributions

XN, JZ, and DH conceived, designed, and directed the project, contributed to the interpretation of the results, and designed the figures. XN and JZ worked out almost all of the technical details, contributed to sample preparation, carried out the experiments, and performed the numerical calculations. XW and SD participated in the acquisition of the data. XS, YZ, and JY partially analyzed the data. All authors provided critical feedback, helped to shape the research, analysis, and manuscript; all discussed the results and contributed to the final version of the manuscript.

## Conflict of Interest

The authors declare that the research was conducted in the absence of any commercial or financial relationships that could be construed as a potential conflict of interest.
