# Supplementary material for: Indigenous Bacteria Have High Potential for Promoting Salix integra Thunb. Remediation of Lead-Contaminated Soil by Adjusting Soil Properties
Source: Front Microbiol. 2020 May 19;11:924. doi: 10.3389/fmicb.2020.00924 (PMC7248224; doi:10.3389/fmicb.2020.00924)
Supplement: Supplementary file 1 [file Data_Sheet_1.docx]

**Supplementary data**

**Table S1.** Effect of different Pb treatments on *salix integra* biomass and Pb uptake

| Treatment | Total biomass(Kg) | Heavy metals concentration in different Organs（mg kg^-1^） | | | | Total absorption per plant（mg） |
| --- | --- | --- | --- | --- | --- | --- |
|  |  | Root | trunk | branch | leave |  |
| CK | 1.15±0.06a | 12.06±0.003c | 1.23±0.002c | 1.02±0.001c | 0.8±0.001c | 3.05±0.007c |
| LT | 1.12±0.03a | 1221.36±111.36b | 60.81±5.36b | 33.5±3.21b | 35.47±2.41b | 362.30±19.85b |
| HT | 0.85±0.08b | 3159.92±138.98a | 128.77±9.25a | 46.33±3.25a | 59.5±4.23a | 717.75±31.23a |

CK, Pb 0 mg kg^-1^; LT, Pb 500 mg kg^-1^; HT, Pb 1500 mg kg^-1^. Different letters indicate that the values differ significantly at p < 0.05. Values represent mean ± SD (n = 3).

| 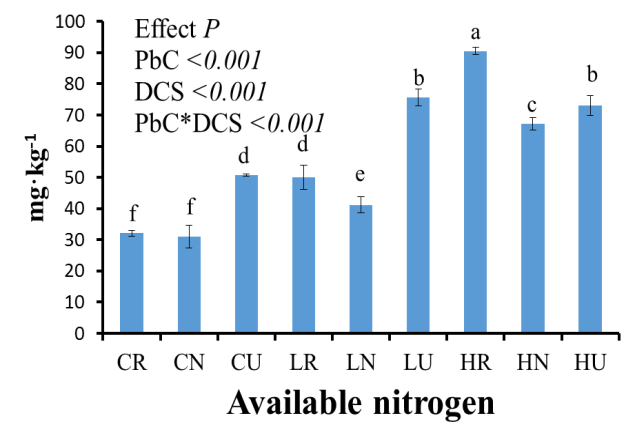  **A** | 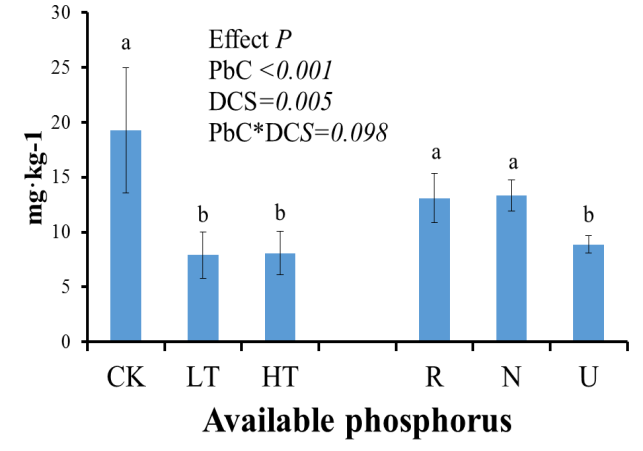  **B** |
| --- | --- |
| 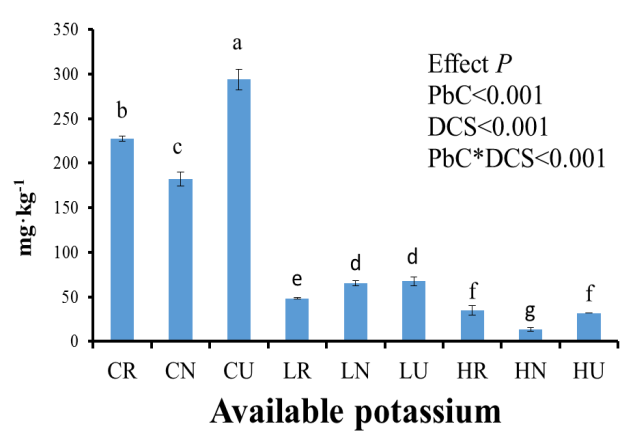  **C** | 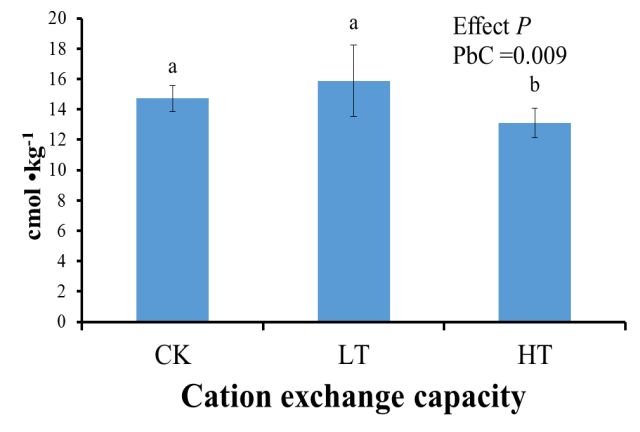  **D** |
| 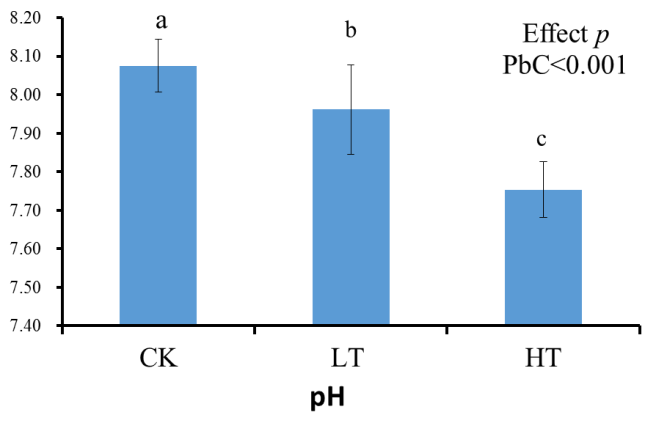  **E** | |
| **FIGURE S1**. Soil properties: **(A)** available nitrogen; **(B)** available phosphorus; **(C)** available potassium; **(D)** cation exchange capacity; and **(E)** pH. CK, Pb 0 mg kg^-1^; LT, Pb 500 mg kg^-1^; HT, Pb 1500 mg kg^-1^; CR, rhizosphere soil in CK; CN, bulk soil in CK; CU, unplanted soil in CK; LR, rhizosphere soil in the low Pb treatment; LN, bulk soil in the low Pb treatment; LU, unplanted soil in the low Pb treatment; HR, rhizosphere soil in the high Pb treatment; HN, bulk soil in the high Pb treatment; HU, unplanted soil in the high Pb treatment. PbC, Pb contamination effect; DCS, soils from different parts of the experiment; PbC*DCS, the interactive effect of Pb contamination and soils from different parts of the experiment. Different letters indicate that the values differ significantly at *p* < 0.05. Values represent mean ± SD (n = 3). | |

| **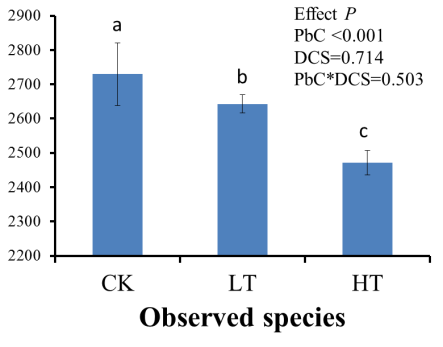**  A | **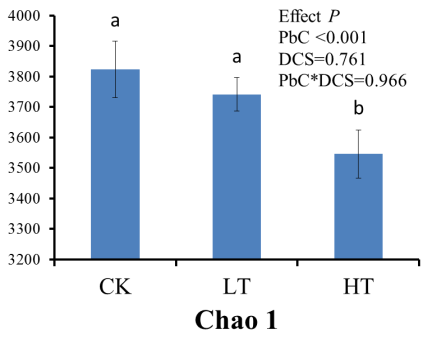** | **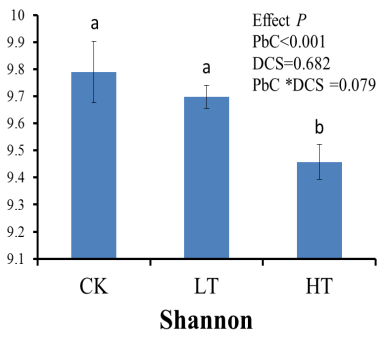** |
| --- | --- | --- |
| 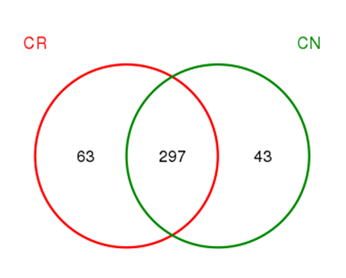  B | 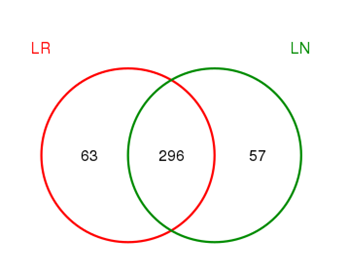 | 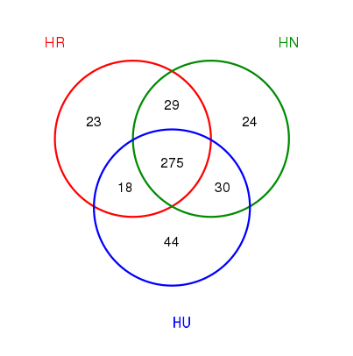 |
| **FIGURE S2**. **(A)** Alpha-diversity of the bacterial community and **(B)** Venn diagram in rhizosphere, bulk, and unplanted soil with different treatments. CK, Pb 0 mg kg^-1^; LT, Pb 500 mg kg^-1^; HT, Pb 1500 mg kg^-1^; CR, rhizosphere soil in CK; CN, bulk soil in CK; LR, rhizosphere soil in the low Pb treatment; LN, bulk soil in the low Pb treatment; HR, rhizosphere soil in the high Pb treatment; HN, bulk soil in the high Pb treatment; HU, unplanted soil in the high Pb treatment. | | |
